# Tracing scientific progress: thematic shifts and emerging directions in proton therapy for glioma based on top-cited papers

**DOI:** 10.3389/fneur.2026.1781410

**Published:** 2026-03-27

**Authors:** Min Liu, Zheng Lv, Qi Li, Yan Zhou, Ning Huang, Shunan Hu

**Affiliations:** 1 Department of Endocrinology, The Central Hospital of Wuhan, Tongji Medical College, Huazhong University of Science and Technology, Wuhan, China; 2 Department of Critical Care Medicine, Union Hospital, Tongji Medical College, Huazhong University of Science and Technology, Wuhan, China; 3 Department of Neurosurgery, Union Hospital, Tongji Medical College, Huazhong University of Science and Technology, Wuhan, China; 4 Department of Neurosurgery, the People’s Hospital of Lichuan City, Lichuan, Hubei, China; 5 Department of Neurosurgery, Xiangyang Central Hospital, Hubei University of Arts and Science, Xiangyang, China

**Keywords:** bibliometric analysis, CiteSpace, glioma, proton therapy, R software, VOSviewer

## Abstract

**Purpose:**

This study aims to delineate the scientific advancements in proton therapy for glioma by analyzing the most frequently cited articles over the past 30 years.

**Methods:**

Web of Science Core Collection and Scopus were independently queried for articles on proton therapy for glioma published between 1997 and 2025. Only items present in both databases were retained for downstream bibliometric analyses. The 100 most-cited articles from this intersection set were extracted for in-depth mapping of countries/regions, institutions, authors, journals, references and keywords. Data processing and visualisation were carried out with CiteSpace, VOSviewer, R-bibliometrix and the online bibliometric platform.

**Results:**

The United States showcased its strong global leadership in this field, leading in publication output and maintaining extensive collaborative networks with multiple countries. Massachusetts General Hospital emerged as the most prolific institution with 29 papers. Tarbell NJ, Macdonald SM, and Yock TI ranked as the top three authors with 15, 15, and 13 papers, respectively. The analysis also revealed emerging research directions such as “Monte-carlo simulations,” “childhood-cancer survivor,” “cognitive function,” “subventricular zone,” and “radiation necrosis,” which warrant increased attention.

**Conclusion:**

This bibliometric analysis represents the first systematic identification of the most influential 100 articles on glioma proton therapy. There is a pressing need to further enhance the clinical efficacy of glioma proton therapy while improving safety and reducing treatment costs, to ultimately bring more benefits to patients.

## 1 Introduction

Gliomas, invasive primary central nervous system tumors with high recurrence rates, challenge oncological treatment (1). Traditional methods like surgery, chemotherapy, and photon radiation therapy show limited efficacy, particularly for high-grade gliomas such as glioblastoma multiforme (GBM) (2, 3). In this context, proton therapy has emerged as a promising radiotherapeutic approach (4, 5). Its unique physical properties, characterized by the Bragg peak phenomenon, allow for precise tumor targeting while sparing surrounding normal brain tissue from excessive radiation exposure (6). This is of paramount importance in the management of gliomas, where the close proximity of critical neurological structures necessitates a delicate balance between tumor control and functional preservation (7). Preclinical and early clinical studies have indicated that proton therapy may offer comparable or superior tumor control rates with a more favorable toxicity profile compared to conventional photon-based radiotherapy (8).

Bibliometrics, an increasingly valuable tool in scientific research evaluation, provides a quantitative and qualitative analysis of academic literature (9, 10). Through sophisticated analytical techniques, bibliometric analysis can uncover the intellectual structure of a field, identify key research clusters, and map the evolution of scientific knowledge over time (11). Hossain et al. (12) conducted a bibliometric analysis to map the research landscape of brain tumor classification using machine learning. Song et al. (13) reported a bibliometric analysis of global research on proton radiotherapy. Despite the rapid expansion of studies focusing on proton therapy for gliomas, no bibliometric analysis specifically targeting gliomas has been published to date.

This is the first comprehensive bibliometric analysis of the literature on proton therapy for gliomas. By elucidating the existing knowledge gaps and highlighting underexplored areas, this analysis can serve as a strategic guide for researchers, clinicians, and policymakers alike, fostering more targeted and efficient allocation of research resources and ultimately advancing the clinical application of proton therapy for gliomas.

## 2 Materials and methods

### 2.1 Dataset selection

The dataset was built from the intersection of two large-scale citation databases—Web of Science Core Collection (WoSCC) and Scopus. On 25 December 2025, identical searches were run in both databases to retrieve records published between 1997 and 2025 whose Title (TI), Abstract (AB) or Author Keywords (AK) contained “proton therapy” AND “glioma” (full query in Supplementary File). Only articles are considered. After deduplication against DOI, title, and abstract using Note Expression software, only articles indexed in both databases were retained. No language restriction was applied. The 100 most-cited articles from this intersection set were downloaded for subsequent bibliometric analyses. This study was conducted in accordance with the PRISMA 2020 statement. The TITAN Guideline 2025 was followed to report any AI-assisted steps (14).

### 2.2 Data visualization and analysis

VOSviewer (version 1.6.16) was utilized to generate visualizations of co-authorship networks (9). In the VOSviewer map, the connections between nodes reflect the relationships between authors or institutions, quantified by total link strength. In the institution collaboration analysis, the normalization method is association strengthen, and the largest set of connected items is displayed. Co-citation analysis of references was conducted using CiteSpace (version 6.3.1) (15). The parameters were set as follows: 2 years per slice, selection criteria (top 50 most cited or frequent items from each slice; g-index: *k* = 15), and pruning (pathfinder). Additional details are provided in the relevant visualization maps. Keyword thematic maps were created using the “bibliometrix” package in R 4.2.0. An online bibliometric analysis platform^1^ was used to illustrate cooperation relationships between countries/regions. Global publication and citation trends were analyzed using Microsoft Excel.

## 3 Results

### 3.1 Trend of publications and citations

**Figure 1:**
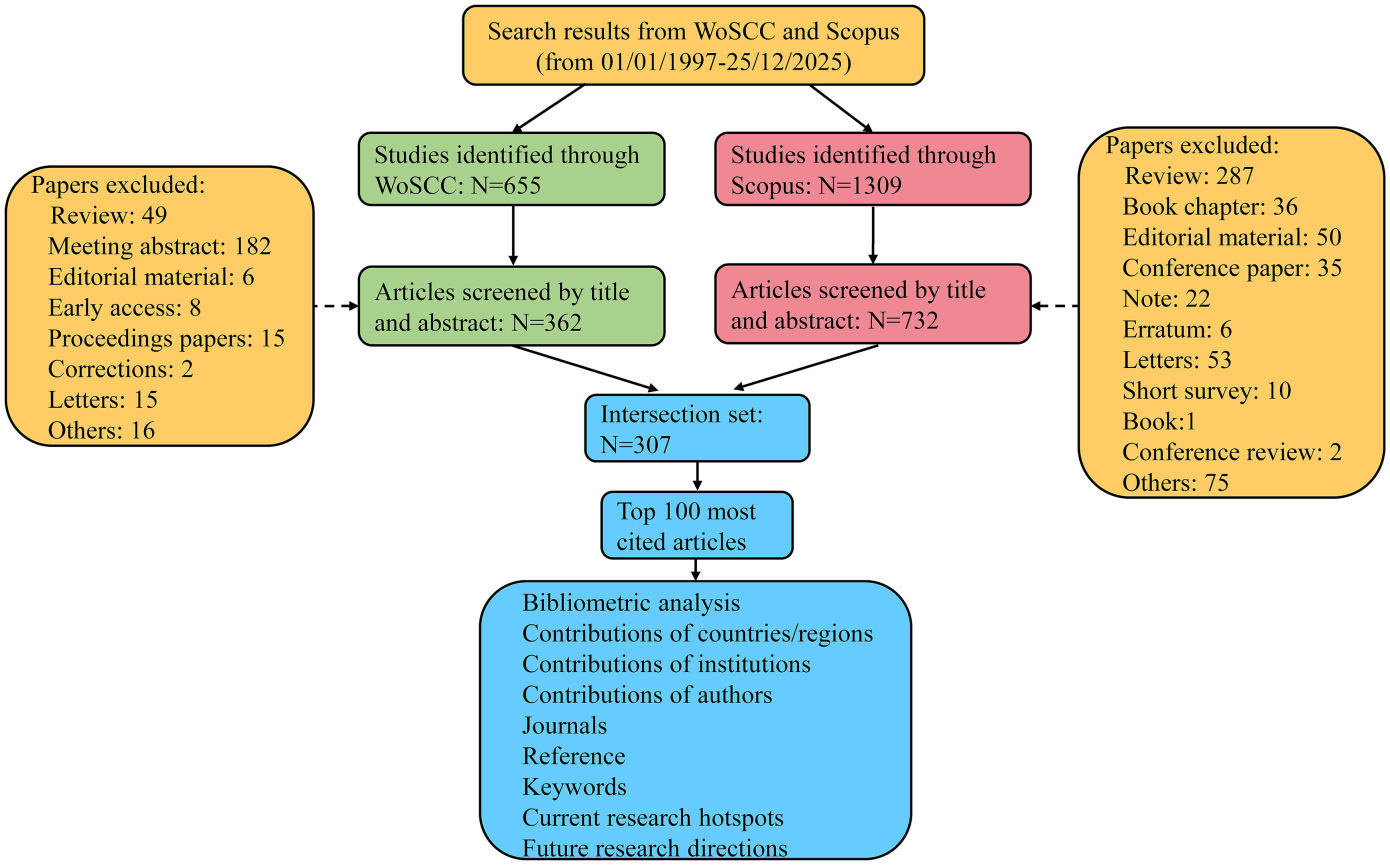
Schematic diagram of the search process.

Between 1997 and 2025, WoSCC and Scopus indexed 655 and 1,309 papers on proton therapy for glioma, respectively (Figure 1). After deduplication and removal of irrelevant records, 307 articles were retained that are jointly covered by both databases. The top 100 most cited articles are listed in the Supplementary Table. These 100 articles garnered between 34 and 301 citations each. Notably, Miralbell et al. (16) stands out with over 300 citations for their work on reducing radiation-induced secondary cancers in pediatric tumors using proton beams. In terms of average annual citations, Kahalley et al. (17) holds the highest with 28.3 citations per year for their study on superior intellectual outcomes in pediatric medulloblastoma patients treated with proton radiotherapy compared to photon radiotherapy.

**Figure 2:**
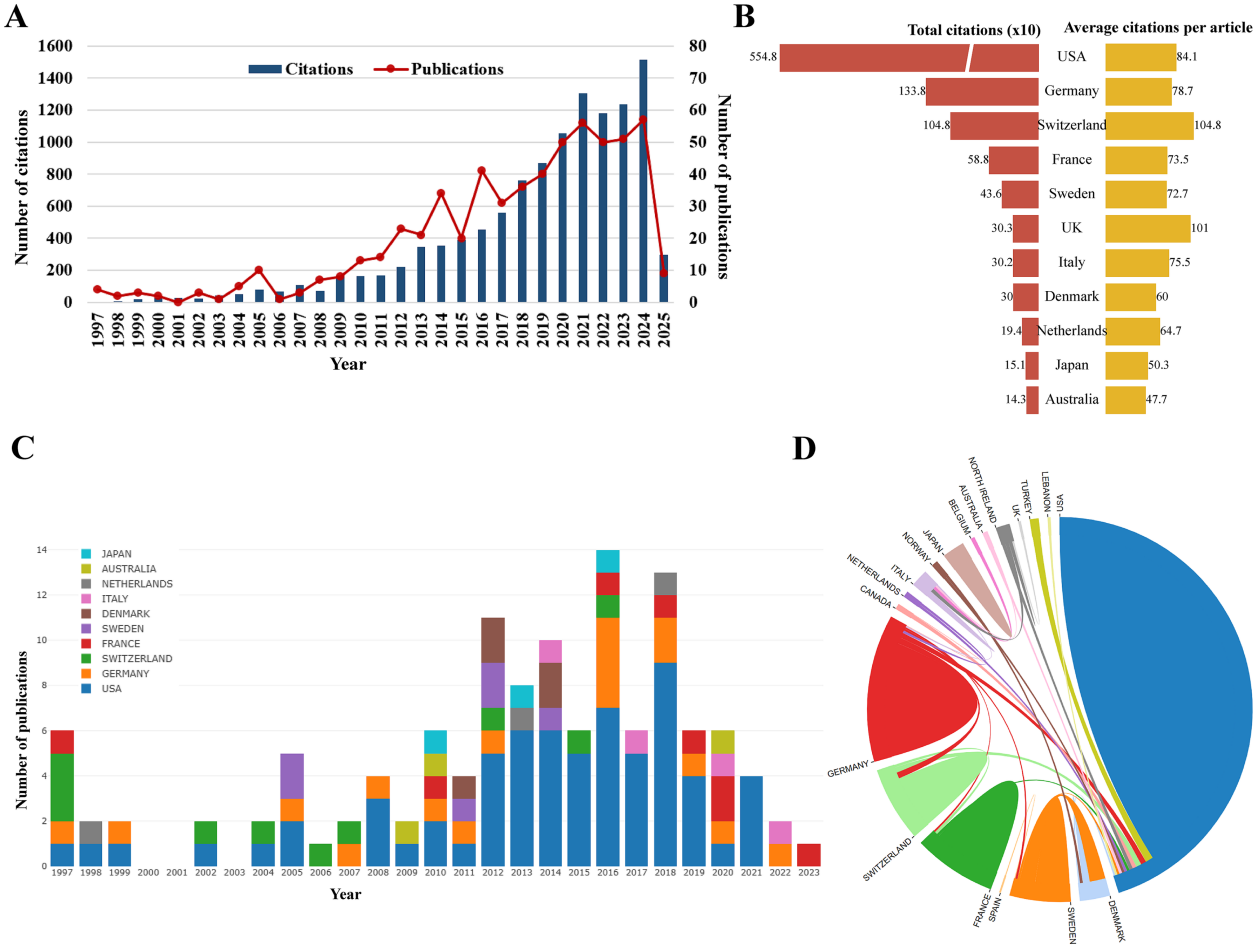
**(A)** Annual counts and total citations of the top 100 most cited articles on glioma proton therapy from 1997 to 2025. **(B)** Total and per-paper average citations of the top 10 countries/regions. **(C)** Annual article output trends of the top 10 countries/regions. **(D)** International collaboration patterns among countries/regions. Each country is represented by a colored segment, with area proportional to its total publication count. Lines between segments denote bilateral co-authorship, and line thickness corresponds to the strengthen of collaboration.

In Figure 2A, the publication and citation trends for glioma proton therapy from 1997 to 2025 are illustrated. Research in this field has gradually increased over time, with a notable surge in recent years. The year 2024 stands out as the most productive and impactful, witnessing the highest number of publications and citations.

### 3.2 Countries/regions and institutions analysis

Figure 2C displays the top 10 countries by the number of publications. The USA leads with 66 articles, significantly more than Germany (17) and Switzerland (10). While the USA has the highest total citations (5,548), Switzerland has the highest average citations per article (Figure 2B). The international collaboration among countries/regions is shown in Figure 2D. This shows that the USA dominance in quantity and overall impact, with Switzerland excelling in per-article influence.

**Figure 3:**
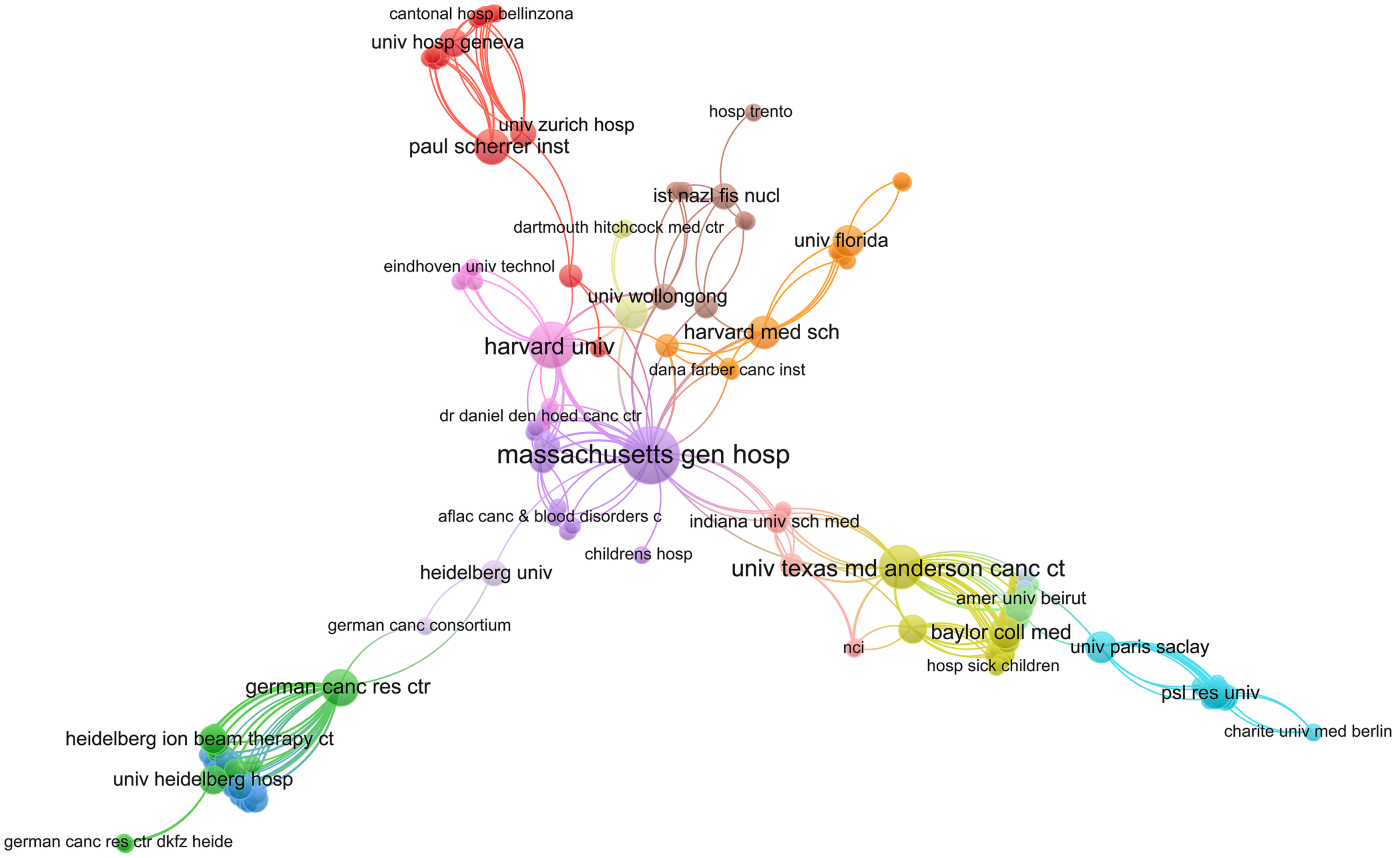
Institutional collaboration network generated by VOSviewer. Node size reflects the number of publications; line thickness indicates collaboration intensity.

Over 160 institutions contributed to these highly cited papers. Massachusetts General Hospital ranked first with 29 articles and 2,457 citations, followed by Harvard University and the University of Texas MD Anderson Cancer Center, which contributed 16 and 13 publications, respectively, (Table 1). In the institutional collaboration network, Massachusetts General Hospital, The University of Texas MD Anderson Cancer Center, Harvard University, and the German Cancer Research Center exhibited the closest collaborative ties (Figure 3).

**Table 1:** The top 10 institutions among the top 100 articles.

Rank	Institutions	Country	Articles	Citations
1	Massachusetts General Hospital	USA	29	2,457
2	Harvard University	USA	16	1,294
3	The University of Texas MD Anderson Cancer Center	USA	13	1,118
4	The German Cancer Research Center	Germany	8	461
5	Paul Scherrer Institute	Switzerland	7	842
6	Loma Linda University	USA	6	492
7	Baylor College of Medicine	USA	6	481
8	Paris Saclay University	France	5	425
9	University of Florida	USA	5	387
10	PSL University	France	5	286

### 3.3 Authors

**Figure 4:**
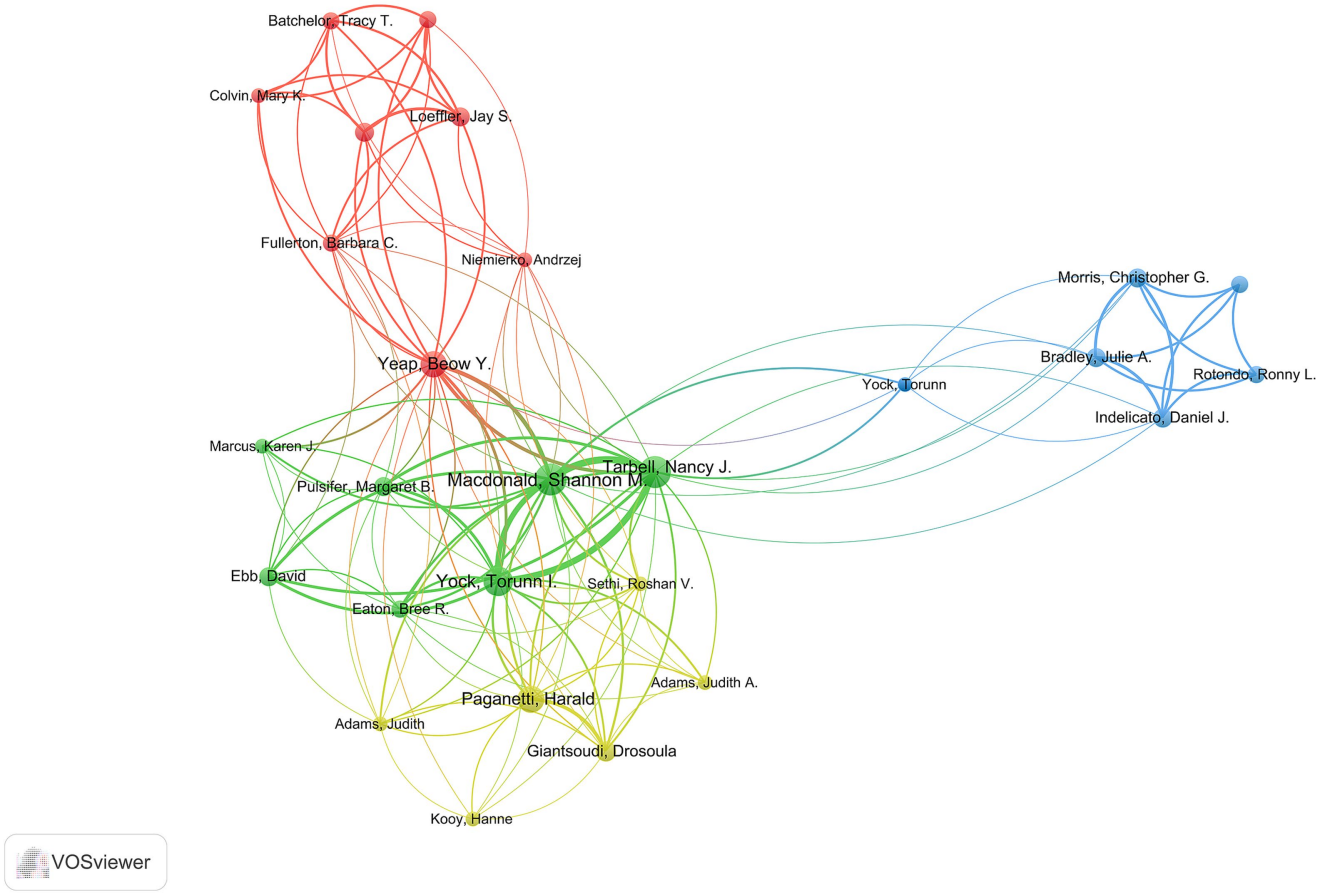
The cooperation network map of authors performed by VOSviewer. The size of nodes represented the number of publications, while the thickness of lines indicated the collaboration strength.

Six hundred and thirty authors contributed to the top 100 highly cited articles on glioma proton therapy. Table 2 lists the top 10 most productive authors. Tarbell NJ, Macdonald SM and Yock TI rank as the top three authors, having published 15, 15, and 13 articles, respectively. Interestingly, while Pulsifer MB from Massachusetts General Hospital has only five articles, these works have achieved the highest citations per article at 102.4, signaling a substantial impact. In terms of co-authorship, as shown in Figure 4, Yeap BY, MacDonald SM, Yock TI, and Paganetti H are positioned at the center of the co-authorship network, boasting the greatest total link strength. These scholars are trailblazers and highly respected authorities in their domain, playing a pivotal role in advancing global proton therapy for glioma research. However, further studies are needed to enhance their expertise in this field.

**Table 2:** The top 10 authors in the field of glioma proton therapy.

Rank	Author	Articles	Citations	Citations per article	Country
1	Tarbell, Nancy J.	15	1,310	87.3	USA
2	Macdonald, Shannon M.	15	1,299	86.6	USA
3	Yock, Torunn I.	13	1,147	88.2	USA
4	Paganetti, Harald	11	783	71.2	USA
5	Yeap, Beow Y.	10	1,020	102	USA
6	Giantsoudi, Drosoula	6	466	77.7	USA
7	Pulsifer, Margaret B.	5	512	102.4	USA
8	Ebb, David	5	417	83.4	USA
9	Indelicato, Daniel J.	5	387	77.4	USA
10	Bradley, Julie A.	5	387	77.4	USA

### 3.4 Contributions of journals

A total of 45 academic journals published top 100 highly cited papers in this research field. There were 61 articles published on the top 10 productive journals (Table 3). International Journal of Radiation Oncology Biologyphysics published the most papers (*n* = 33, IF 2024 = 6.5), followed by Radiotherapy and Oncology (*n* = 9, IF 2024 = 5.3) and Acta Oncologica (*n* = 8, IF 2024 = 2.2).

**Table 3:** The distribution of top 10 journals.

Rank	Journal	Article	Citation	Citation per article	IF 2024	JCR 2024
1	International Journal of Radiation Oncology Biologyphysics	33	2,763	83.7	6.5	Q1
2	Radiotherapy and Oncology	9	674	74.9	5.3	Q1
3	Acta Oncologica	8	617	77.1	2.2	Q3
4	Neuro Oncology	7	539	77	13.4	Q1
5	Cancer	6	412	68.7	5.1	Q1
6	Strahlentherapie Undonkologie	5	339	67.8	2.5	Q2
7	Physics In Medicine Andbiology	6	410	68.3	3.4	Q1
8	Journal of Neurooncology	4	187	46.8	3.1	Q2
9	Radiation Oncology	4	224	56	3.2	Q1
10	Scientific Reports	4	297	74.3	3.9	Q1

IF, Impact Factor. JCR, Journal Citation Reports.

### 3.5 Co-cited references

**Figure 5:**
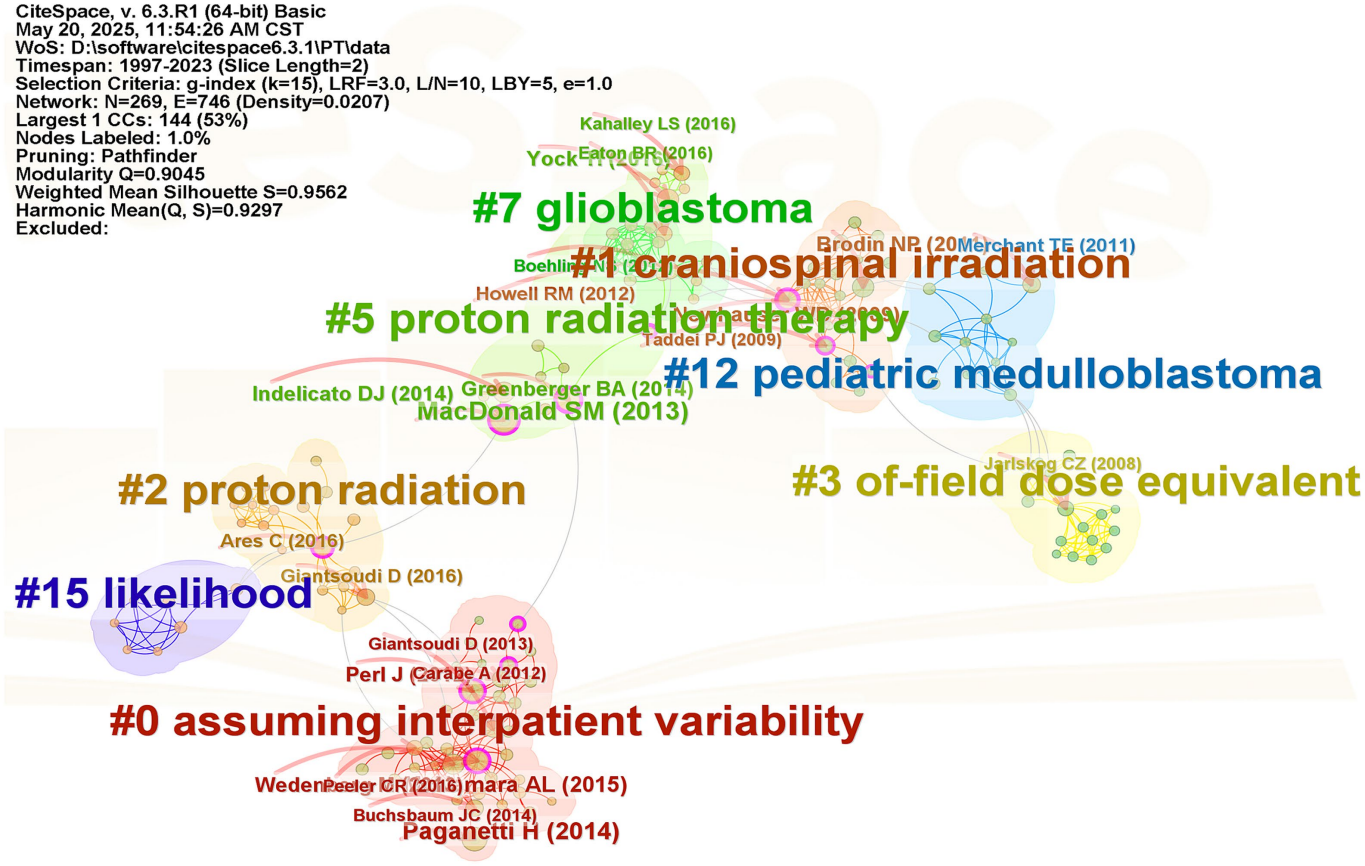
Network visualization map of references co-citation analysis performed by CiteSpace. Eight labeled clusters are colored. The nodes on the line represented the co-citated references.

Reference co-citation analysis was employed to uncover the predominant research themes in glioma proton therapy. As shown in Figure 5, nodes symbolize cited references. The modularity *Q* value of 0.9045 and the mean Silhouette *S* value of 0.9562 indicate impressive clustering effect. The analysis yielded eight distinct clusters. In this network, betweenness centrality, marked in purple, was used to evaluate the importance of nodes. This measure reveals the role of references in connecting different parts of the network, helping to identify key studies that have significantly shaped the field’s research landscape.

### 3.6 Thematic map of keywords analysis

**Figure 6:**
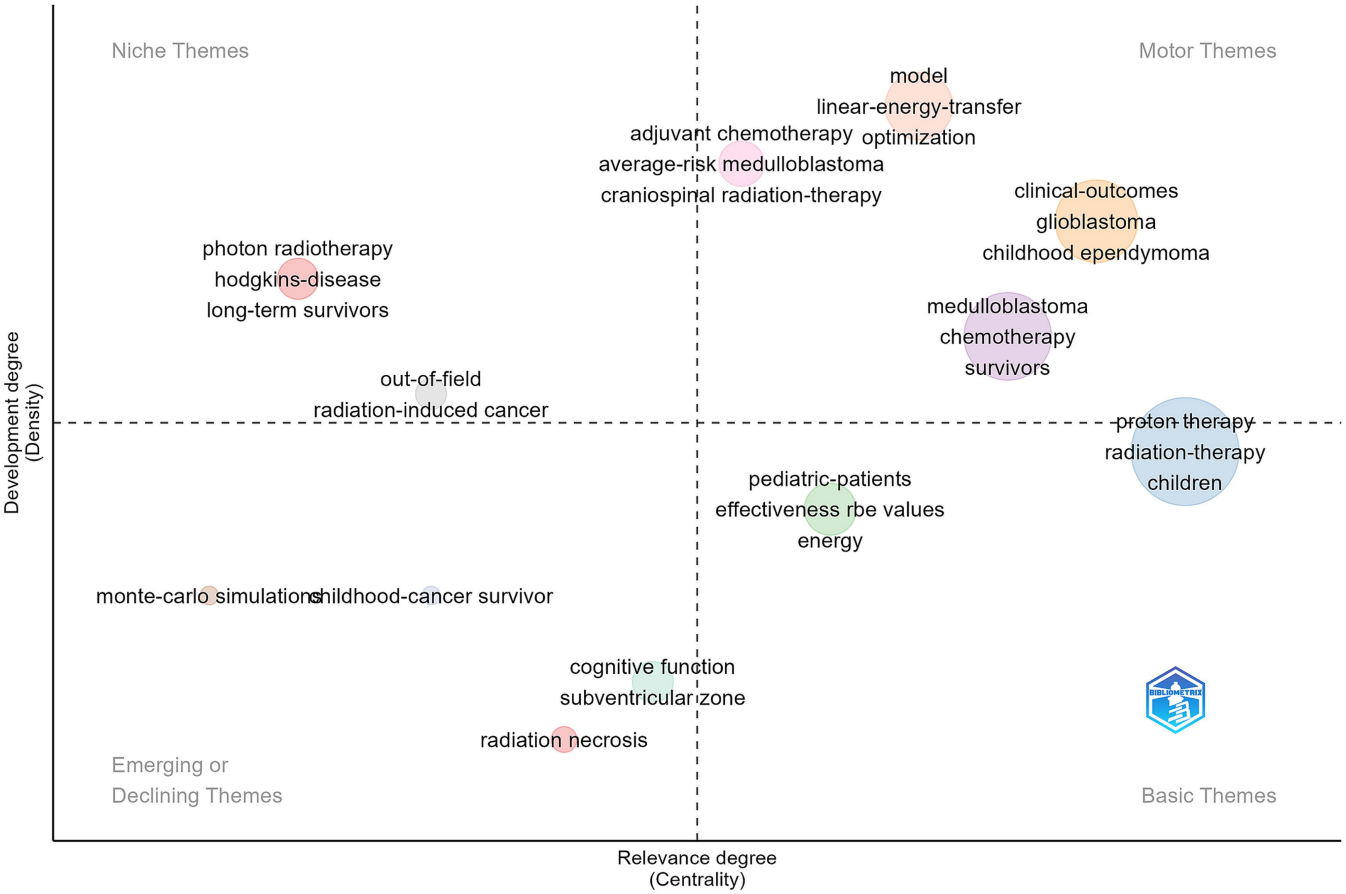
The thematic map based on keywords performed by “bibliometrix” R language package. The *x*-axis represents the relevance degree (centrality) of each theme, indicating its importance within the field, while the *y*-axis represents the development degree (density), reflecting the maturity and internal connectivity of research on that theme. The size of each bubble is proportional to the number of publications associated with the corresponding theme.

The thematic map of keywords is used to illustrate the connections between different themes and the intensity of these connections within a theme, thereby highlighting their significance and progression in the field of glioma proton therapy. Centrality (horizontal axis) reflects a theme’s relevance to other themes, with higher centrality indicating greater significance in the research area. Density (vertical axis) reflects the strength of the relationship among keywords within a theme, with higher density suggesting a more developed or mature concept. Examples of emerging or declining themes include “monte-carlo simulations,” “childhood-cancer survivor,” “cognitive function,” “subventricular zone,” and “radiation necrosis” (Figure 6). The quantitative indicators were concluded in the Supplementary Table 1. By understanding the landscape of existing research, scholars can strategically allocate research resources and identify gaps where further investigation is needed.

## 4 Discussion

Significant studies with groundbreaking contributions are usually the most cited in a field. Bibliometric analysis offers an intuitive way to grasp research progress, and highly cited papers are highly valuable for future research. This study presents an overview of glioma proton therapy based on the top 100 most cited articles, emphasizing hotspots and research trends to guide future studies.

Globally, the USA holds a predominant position in this research domain, boasting the majority of highly cited articles (*n* = 66). All of the top 10 most productive authors are affiliated with American institutions, and the USA engages in the most frequent collaborations with other countries/regions. Notably, scholars Tarbell NJ and Macdonald SM from Massachusetts General Hospital have maintained a close collaborative relationship, ranking first and second with 15 highly cited documents each. Their research primarily centers on proton therapy for pediatric malignancies, including medulloblastoma, pediatric craniopharyngioma, and Ewing’s sarcoma (18–20). They emphasize the clinical efficacy of proton therapy and its late side effects in pediatric patients, such as whether craniospinal proton radiotherapy induces growth retardation (21). Enhancing the efficacy of proton therapy while reducing its impact on neurological function remains a pivotal research objective. Keywords are used to understand how a field has developed. From the thematic map (Figure 6), we can predict that “monte-carlo simulations,” “childhood-cancer survivor,” “cognitive function,” “subventricular zone” and “radiation necrosis” as the emerging research directions should be given more consideration.

Radiation necrosis, defined as the irreversible damage and death of healthy tissue induced by radiotherapy, represents a long-term CNS complication that can emerge months or even years post-treatment (22). Monte Carlo simulations offer a promising solution by enabling more precise predictions of proton beam behavior within the human body (23). This facilitates the optimized design of complex intensity-modulated fields, enhances the precision of treatment planning, and reduces the radiation dose to surrounding healthy tissues. Moreover, Monte Carlo simulations can integrate various factors, including proton beam uncertainties and the heterogeneous anatomy and tissue density of patients, thereby minimizing uncertainties during treatment and improving its safety and reliability (24). By refining the radiation strategy for lesions, the risk of radiation necrosis can be mitigated. Multi-center randomized controlled trials should be conducted to validate whether optimized proton treatment plans using Monte Carlo simulations can significantly reduce the incidence of radiation necrosis.

The subventricular zone (SVZ) is a region in the brain with the highest concentration of neural stem cell (NSC) (25). Molecular mechanisms involving cytoskeletal proteins, telomerase, tumor suppressor proteins, transcription factors, and growth factors within NSC of the SVZ can drive the development of GBM (26). While radiotherapy targeting this region may prevent the progression of primary and recurrent GBM, it is not without risk (27). Irradiation of larger brain volumes can lead to significant adverse reactions (28). Dose-escalation trials should be designed to explore the feasibility of moderate irradiation to this region for preventing glioma recurrence while preserving neural cells.

This study has certain limitations. Despite being the most commonly used for analyzing highly cited literatures in a specific field, the citation-based selection might have omitted some recent and impactful studies, potentially missing out on some emerging trends. We encourage readers to consider this when interpreting our findings and to stay informed of the latest findings in the field.

## 5 Conclusion

Proton therapy has shown significant potential in the treatment of glioma, yet several challenges and uncertainties remain in its application. Unlike photon therapy, which is more established, proton therapy for glioma requires further advancement and optimization to fully realize its benefits. Pediatric oncology has already witnessed substantial advantages from proton therapy, mainly attributed to its lesser impact on children’s developing organs. Looking to the future, ongoing technical innovations and expanded clinical applications are expected to lower treatment costs and reduce uncertainties.

## Data Availability

The original contributions presented in the study are included in the article/Supplementary material, further inquiries can be directed to the corresponding authors.

## Footnotes

1
https://bibliometric.com/
