# Supplementary material for: Tracing scientific progress: thematic shifts and emerging directions in proton therapy for glioma based on top-cited papers
Source: Front Neurol. 2026 Mar 27;17:1781410. doi: 10.3389/fneur.2026.1781410 (PMC13065506; doi:10.3389/fneur.2026.1781410)
Supplement: Supplementary file 1 [file Table_1.docx]

Search strategy:

“#1: TI/AB/AK= “glioma*” OR “astrocytoma” OR “glioblastoma*” OR “ependymoma*” OR “ganglioglioma” OR “gliosarcoma” OR “medulloblastoma” OR “oligodendroglioma” OR “GBM” OR “oligoastrocytoma” OR “glial cell tumor*”.

#2: TI/AB/AK= “proton radiotherap*” OR “proton beam radiotherap*” OR “proton irradiation” OR “proton beam irradiation” OR “proton radiation therap*” OR “proton beam radiation therap*” OR “proton therap*” OR “proton beam therap*”.

#1 AND #2”
