# Supplementary material for: Tracing scientific progress: thematic shifts and emerging directions in proton therapy for glioma based on top-cited papers
Source: Front Neurol. 2026 Mar 27;17:1781410. doi: 10.3389/fneur.2026.1781410 (PMC13065506; doi:10.3389/fneur.2026.1781410)
Supplement: Supplementary file 2 [file Table_2.docx]

The top 100 cited articles on proton therapy for glioma

| Rank | Title | Journal | Year | TC | AC/Y |
| --- | --- | --- | --- | --- | --- |
| 1 | Potential reduction of the incidence of radiation-induced second cancers by using proton beams in the treatment of pediatric tumors | INTERNATIONAL JOURNAL OF RADIATION ONCOLOGY BIOLOGY PHYSICS | 2002 | 301 | 12.54 |
| 2 | Proton versus photon radiotherapy for common pediatric brain tumors: Comparison of models of dose characteristics and their relationship to cognitive function | PEDIATRIC BLOOD & CANCER | 2008 | 245 | 13.61 |
| 3 | Long-term toxic eff ects of proton radiotherapy for paediatric medulloblastoma: a phase 2 single-arm study | LANCET ONCOLOGY | 2016 | 242 | 24.2 |
| 4 | Treatment planning and verification of proton therapy using spot scanning: Initial experiences | MEDICAL PHYSICS | 2004 | 234 | 10.64 |
| 5 | Clinical evidence of variable proton biological effectiveness in pediatric patients treated for ependymoma | RADIOTHERAPY AND ONCOLOGY | 2016 | 216 | 21.6 |
| 6 | Relative Biological Effectiveness Variation Along Monoenergetic and Modulated Bragg Peaks of a 62-MeV Therapeutic Proton Beam: A Preclinical Assessment | INTERNATIONAL JOURNAL OF RADIATION ONCOLOGY BIOLOGY PHYSICS | 2014 | 188 | 15.67 |
| 7 | Treatment planning with protons for pediatric retinoblastoma, medulloblastoma, and pelvic sarcoma: How do protons compare with other conformal techniques? | INTERNATIONAL JOURNAL OF RADIATION ONCOLOGY BIOLOGY PHYSICS | 2005 | 186 | 8.86 |
| 8 | Radiation necrosis versus glioma recurrence: Conventional MR imaging clues to diagnosis | AMERICAN JOURNAL OF NEURORADIOLOGY | 2005 | 185 | 8.81 |
| 9 | Superior Intellectual Outcomes After Proton Radiotherapy Compared With Photon Radiotherapy for Pediatric Medulloblastoma | JOURNAL OF CLINICAL ONCOLOGY | 2020 | 170 | 28.33 |
| 10 | Incidence and dosimetric parameters of pediatric brainstem toxicity following proton therapy | ACTA ONCOLOGICA | 2014 | 159 | 13.25 |
| 11 | Reoptimization of Intensity Modulated Proton Therapy Plans Based on Linear Energy Transfer | INTERNATIONAL JOURNAL OF RADIATION ONCOLOGY BIOLOGY PHYSICS | 2016 | 154 | 15.4 |
| 12 | Proton radiotherapy for childhood ependymoma: Initial clinical outcomes and dose comparisons | INTERNATIONAL JOURNAL OF RADIATION ONCOLOGY BIOLOGY PHYSICS | 2008 | 154 | 8.56 |
| 13 | Conformal proton radiation therapy for pediatric low-grade astrocytomas | STRAHLENTHERAPIE UND ONKOLOGIE | 2002 | 129 | 5.38 |
| 14 | Proton beam radiation induces DNA damage and cell apoptosis in glioma stem cells through reactive oxygen species | SCIENTIFIC REPORTS | 2015 | 118 | 10.73 |
| 15 | Endocrine outcomes with proton and photon radiotherapy for standard risk medulloblastoma | NEURO-ONCOLOGY | 2016 | 116 | 11.6 |
| 16 | Glioma Recurrence Versus Radiation Necrosis? A Pilot Comparison of Arterial Spin-Labeled, Dynamic Susceptibility Contrast Enhanced MRI, and FDG-PET Imaging | ACADEMIC RADIOLOGY | 2010 | 116 | 7.25 |
| 17 | Late Contrast Enhancing Brain Lesions in Proton-Treated Patients With Low-Grade Glioma: Clinical Evidence for Increased Periventricular Sensitivity and Variable RBE | INTERNATIONAL JOURNAL OF RADIATION ONCOLOGY BIOLOGY PHYSICS | 2020 | 113 | 18.83 |
| 18 | Proton radiotherapy for pediatric central nervous system ependymoma: clinical outcomes for 70 patients | NEURO-ONCOLOGY | 2013 | 112 | 8.62 |
| 19 | Proton radiation therapy (PRT) for pediatric optic pathway gliomas: Comparison with 3D planned conventional photons and a standard photon technique | INTERNATIONAL JOURNAL OF RADIATION ONCOLOGY BIOLOGY PHYSICS | 1999 | 108 | 4 |
| 20 | Incidence of CNS Injury for a Cohort of 111 Patients Treated With Proton Therapy for Medulloblastoma: LET and RBE Associations for Areas of Injury | INTERNATIONAL JOURNAL OF RADIATION ONCOLOGY BIOLOGY PHYSICS | 2016 | 104 | 10.4 |
| 21 | Proton Therapy for Low-Grade Gliomas: Results From a Prospective Trial | CANCER | 2015 | 103 | 9.36 |
| 22 | Radiobiological risk estimates of adverse events and secondary cancer for proton and photon radiation therapy of pediatric medulloblastoma | ACTA ONCOLOGICA | 2011 | 102 | 6.8 |
| 23 | Reducing toxicity from craniospinal irradiation: Using proton beams to treat medulloblastoma in young children | CANCER JOURNAL | 2004 | 102 | 4.64 |
| 24 | Imaging Changes in Pediatric Intracranial Ependymoma Patients Treated With Proton Beam Radiation Therapy Compared to Intensity Modulated Radiation Therapy | INTERNATIONAL JOURNAL OF RADIATION ONCOLOGY BIOLOGY PHYSICS | 2015 | 101 | 9.18 |
| 25 | Differential DNA repair pathway choice in cancer cells after proton- and photon-irradiation | RADIOTHERAPY AND ONCOLOGY | 2015 | 100 | 9.09 |
| 26 | Site-specific range uncertainties caused by dose calculation algorithms for proton therapy | PHYSICS IN MEDICINE AND BIOLOGY | 2014 | 99 | 8.25 |
| 27 | Outcomes Following Proton Therapy for Pediatric Low-Grade Glioma | INTERNATIONAL JOURNAL OF RADIATION ONCOLOGY BIOLOGY PHYSICS | 2019 | 94 | 13.43 |
| 28 | Proton therapy reduces the likelihood of high-grade radiation-induced lymphopenia in glioblastoma patients: phase II randomized study of protons vs photons | NEURO-ONCOLOGY | 2021 | 92 | 18.4 |
| 29 | Dosimetric advantages of proton therapy over conventional radiotherapy with photons in young patients and adults with low-grade glioma | STRAHLENTHERAPIE UND ONKOLOGIE | 2016 | 91 | 9.1 |
| 30 | Cost-effectiveness of proton radiation in the treatment of childhood medulloblastoma | CANCER | 2005 | 91 | 4.33 |
| 31 | Proton minibeam radiation therapy widens the therapeutic index for high-grade gliomas | SCIENTIFIC REPORTS | 2018 | 88 | 11 |
| 32 | Does electron and proton therapy reduce the risk of radiation induced cancer after spinal irradiation for childhood medulloblastoma? A comparative treatment planning study | ACTA ONCOLOGICA | 2005 | 87 | 4.14 |
| 33 | Pediatric medulloblastoma: Radiation treatment technique and patterns of failure | INTERNATIONAL JOURNAL OF RADIATION ONCOLOGY BIOLOGY PHYSICS | 1997 | 85 | 2.93 |
| 34 | Assessment of radiation-induced second cancer risks in proton therapy and IMRT for organs inside the primary radiation field | PHYSICS IN MEDICINE AND BIOLOGY | 2012 | 84 | 6 |
| 35 | Prospective Study of Health-Related Quality of Life for Children With Brain Tumors Treated With Proton Radiotherapy | JOURNAL OF CLINICAL ONCOLOGY | 2012 | 83 | 5.93 |
| 36 | Temporal lobe (TL) damage following surgery and high-dose photon and proton irradiation in 96 patients affected by chordomas and chondrosarcomas of the base of the skull | INTERNATIONAL JOURNAL OF RADIATION ONCOLOGY BIOLOGY PHYSICS | 1998 | 83 | 2.96 |
| 37 | Comparison of therapeutic dosimetric data from passively scattered proton and photon craniospinal irradiations for medulloblastoma | RADIATION ONCOLOGY | 2012 | 81 | 5.79 |
| 38 | Early Cognitive Outcomes Following Proton Radiation in Pediatric Patients With Brain and Central Nervous System Tumors | INTERNATIONAL JOURNAL OF RADIATION ONCOLOGY BIOLOGY PHYSICS | 2015 | 80 | 7.27 |
| 39 | Heidelberg Ion Therapy Center (HIT): Initial clinical experience in the first 80 patients | ACTA ONCOLOGICA | 2010 | 78 | 4.88 |
| 40 | Tumor Control in RG2 Glioma-Bearing Rats: A Comparison Between Proton Minibeam Therapy and Standard Proton Therapy | INTERNATIONAL JOURNAL OF RADIATION ONCOLOGY BIOLOGY PHYSICS | 2019 | 77 | 11 |
| 41 | Potential role of proton therapy in the treatment of pediatric medulloblasatoma primitive neuro-ectodermal tumors: Spinal theca irradiation | INTERNATIONAL JOURNAL OF RADIATION ONCOLOGY BIOLOGY PHYSICS | 1997 | 73 | 2.52 |
| 42 | A prospective phase II randomized trial of proton radiotherapy vs intensity-modulated radiotherapy for patients with newly diagnosed glioblastoma | NEURO-ONCOLOGY | 2021 | 71 | 14.2 |
| 43 | A comparative study on the risks of radiogenic second cancers and cardiac mortality in a set of pediatric medulloblastoma patients treated with photon or proton craniospinal irradiation | RADIOTHERAPY AND ONCOLOGY | 2014 | 71 | 5.92 |
| 44 | Phase I/II trial of hyperfractionated concomitant boost proton radiotherapy for supratentorial glioblastoma multiforme | INTERNATIONAL JOURNAL OF RADIATION ONCOLOGY BIOLOGY PHYSICS | 2010 | 71 | 4.44 |
| 45 | Low early ototoxicity rates for pediatric medulloblastoma patients treated with proton radiotherapy | RADIATION ONCOLOGY | 2011 | 68 | 4.53 |
| 46 | Brainstem Injury in Pediatric Patients With Posterior Fossa Tumors Treated With Proton Beam Therapy and Associated Dosimetric Factors | INTERNATIONAL JOURNAL OF RADIATION ONCOLOGY BIOLOGY PHYSICS | 2018 | 66 | 8.25 |
| 47 | Comparison of risk of radiogenic second cancer following photon and proton craniospinal irradiation for a pediatric medulloblastoma patient | PHYSICS IN MEDICINE AND BIOLOGY | 2013 | 66 | 5.08 |
| 48 | LET-weighted doses effectively reduce biological variability in proton radiotherapy planning | PHYSICS IN MEDICINE AND BIOLOGY | 2018 | 65 | 8.13 |
| 49 | Assessment of out-of-field absorbed dose and equivalent dose in proton fields | MEDICAL PHYSICS | 2010 | 64 | 4 |
| 50 | Clinically Applicable Monte Carlo-based Biological Dose Optimization for the Treatment of Head and Neck Cancers With Spot-Scanning Proton Therapy | INTERNATIONAL JOURNAL OF RADIATION ONCOLOGY BIOLOGY PHYSICS | 2016 | 62 | 6.2 |
| 51 | Neurocognitive effects of proton radiation therapy in adults with low-grade glioma | JOURNAL OF NEURO-ONCOLOGY | 2016 | 61 | 6.1 |
| 52 | Patterns of Failure After Proton Therapy in Medulloblastoma: Linear Energy Transfer Distributions and Relative Biological Effectiveness Associations for Relapses | INTERNATIONAL JOURNAL OF RADIATION ONCOLOGY BIOLOGY PHYSICS | 2014 | 61 | 5.08 |
| 53 | Proton radiography and proton computed tomography based on time-resolved dose measurements | PHYSICS IN MEDICINE AND BIOLOGY | 2013 | 61 | 4.69 |
| 54 | Long-term outcomes and late adverse effects of a prospective study on proton radiotherapy for patients with low-grade glioma | RADIOTHERAPY AND ONCOLOGY | 2019 | 60 | 8.57 |
| 55 | Outcomes following proton therapy for pediatric ependymoma | ACTA ONCOLOGICA | 2018 | 60 | 7.5 |
| 56 | Cost Effectiveness of Proton Therapy Compared With Photon Therapy in the Management of Pediatric Medulloblastoma | CANCER | 2013 | 59 | 4.54 |
| 57 | Cognitive and Adaptive Outcomes After Proton Radiation for Pediatric Patients With Brain Tumors | INTERNATIONAL JOURNAL OF RADIATION ONCOLOGY BIOLOGY PHYSICS | 2018 | 58 | 7.25 |
| 58 | Use of proton therapy for re-irradiation in pediatric intracranial ependymoma | RADIOTHERAPY AND ONCOLOGY | 2015 | 57 | 5.18 |
| 59 | A Comparison of Critical Structure Dose and Toxicity Risks in Patients with Low Grade Gliomas Treated with IMRT versus Proton Radiation Therapy | TECHNOLOGY IN CANCER RESEARCH & TREATMENT | 2013 | 57 | 4.38 |
| 60 | Life years lost-comparing potentially fatal late complications after radiotherapy for pediatric medulloblastoma on a common scale | CANCER | 2012 | 57 | 4.07 |
| 61 | Estimated clinical benefit of protecting neurogenesis in the developing brain during radiation therapy for pediatric medulloblastoma | NEURO-ONCOLOGY | 2012 | 57 | 4.07 |
| 62 | Short and long-term evaluation of the impact of proton minibeam radiation therapy on motor, emotional and cognitive functions | SCIENTIFIC REPORTS | 2020 | 56 | 9.33 |
| 63 | Progression-Free Survival of Children With Localized Ependymoma Treated With Intensity-Modulated Radiation Therapy or Proton-Beam Radiation Therapy | CANCER | 2017 | 54 | 6 |
| 64 | Potential role of proton therapy in the treatment of pediatric medulloblastoma primitive neuroectodermal tumors: Reduction of the supratentorial target volume | INTERNATIONAL JOURNAL OF RADIATION ONCOLOGY BIOLOGY PHYSICS | 1997 | 52 | 1.79 |
| 65 | Photon vs. proton radiochemotherapy: Effects on brain tissue volume and perfusion | RADIOTHERAPY AND ONCOLOGY | 2018 | 50 | 6.25 |
| 66 | Proton Radiation Therapy for Pediatric Medulloblastoma and Supratentorial Primitive Neuroectodermal Tumors: Outcomes for Very Young Children Treated With Upfront Chemotherapy | INTERNATIONAL JOURNAL OF RADIATION ONCOLOGY BIOLOGY PHYSICS | 2013 | 50 | 3.85 |
| 67 | Dilemmas concerning dose distribution and the influence of relative biological effect in proton beam therapy of medulloblastoma | BRITISH JOURNAL OF RADIOLOGY | 2012 | 50 | 3.57 |
| 68 | Prospective, longitudinal comparison of neurocognitive change in pediatric brain tumor patients treated with proton radiotherapy versus surgery only | NEURO-ONCOLOGY | 2019 | 49 | 7 |
| 69 | Socioeconomic Factors Affect the Selection of Proton Radiation Therapy for Children | CANCER | 2017 | 48 | 5.33 |
| 70 | Proton Treatment Techniques for Posterior Fossa Tumors: Consequences for Linear Energy Transfer and Dose-Volume Parameters for the Brainstem and Organs at Risk | INTERNATIONAL JOURNAL OF RADIATION ONCOLOGY BIOLOGY PHYSICS | 2017 | 47 | 5.22 |
| 71 | Can particle beam therapy be improved using helium ions? - a planning study focusing on pediatric patients | ACTA ONCOLOGICA | 2016 | 46 | 4.6 |
| 72 | Proton therapy for pediatric cranial tumors: Preliminary report on treatment and disease-related morbidities | INTERNATIONAL JOURNAL OF RADIATION ONCOLOGY BIOLOGY PHYSICS | 1997 | 46 | 1.59 |
| 73 | Pencil beam scanning proton therapy for pediatric intracranial ependymoma | JOURNAL OF NEURO-ONCOLOGY | 2016 | 45 | 4.5 |
| 74 | Radiation induced contrast enhancement after proton beam therapy in patients with low grade glioma - How safe are protons? | RADIOTHERAPY AND ONCOLOGY | 2022 | 44 | 11 |
| 75 | Radiosensitivity of Patient-Derived Glioma Stem Cell 3-Dimensional Cultures to Photon, Proton, and Carbon Irradiation | INTERNATIONAL JOURNAL OF RADIATION ONCOLOGY BIOLOGY PHYSICS | 2016 | 44 | 4.4 |
| 76 | Out-of-field dose equivalents delivered by passively scattered therapeutic proton beams for clinically relevant field configurations | INTERNATIONAL JOURNAL OF RADIATION ONCOLOGY BIOLOGY PHYSICS | 2009 | 44 | 2.59 |
| 77 | Relating the proton relative biological effectiveness to tumor control and normal tissue complication probabilities assuming interpatient variability in α/β | ACTA ONCOLOGICA | 2017 | 43 | 4.78 |
| 78 | Intensity-modulated proton therapy, volumetric-modulated arc therapy, and 3D conformal radiotherapy in anaplastic astrocytoma and glioblastoma | STRAHLENTHERAPIE UND ONKOLOGIE | 2016 | 42 | 4.2 |
| 79 | Estimated risk of radiation-induced cancer following paediatric cranio-spinal irradiation with electron, photon and proton therapy | ACTA ONCOLOGICA | 2014 | 42 | 3.5 |
| 80 | Hippocampal sparing radiotherapy for pediatric medulloblastoma: impact of treatment margins and treatment technique | NEURO-ONCOLOGY | 2014 | 42 | 3.5 |
| 81 | Increase of pseudoprogression and other treatment related effects in low-grade glioma patients treated with proton radiation and temozolomide | JOURNAL OF NEURO-ONCOLOGY | 2019 | 41 | 5.86 |
| 82 | Ototoxicity and cochlear sparing in children with medulloblastoma: Proton vs. photon radiotherapy | RADIOTHERAPY AND ONCOLOGY | 2018 | 41 | 5.13 |
| 83 | Dosimetric Comparison of Proton Radiation Therapy, Volumetric Modulated Arc Therapy, and Three-Dimensional Conformal Radiotherapy Based on Intracranial Tumor Location | CANCERS | 2018 | 40 | 5 |
| 84 | Risk of Radiation Vasculopathy and Stroke in Pediatric Patients Treated With Proton Therapy for Brain and Skull Base Tumors | INTERNATIONAL JOURNAL OF RADIATION ONCOLOGY BIOLOGY PHYSICS | 2018 | 40 | 5 |
| 85 | Radiation-Induced Large Vessel Cerebral Vasculopathy in Pediatric Patients With Brain Tumors Treated With Proton Radiation Therapy | INTERNATIONAL JOURNAL OF RADIATION ONCOLOGY BIOLOGY PHYSICS | 2017 | 40 | 4.44 |
| 86 | Proton beam therapy with concurrent chemotherapy for glioblastoma multiforme: comparison of nimustine hydrochloride and temozolomide | JOURNAL OF NEURO-ONCOLOGY | 2016 | 40 | 4 |
| 87 | Reirradiation for recurrent malignant brain tumor with radiotherapy or proton beam therapy Technical considerations based on experience at a single institution | STRAHLENTHERAPIE UND ONKOLOGIE | 2013 | 40 | 3.08 |
| 88 | First experiences in treatment of low-grade glioma grade I and II with proton therapy | RADIATION ONCOLOGY | 2012 | 40 | 2.86 |
| 89 | A Multi-institutional Comparative Analysis of Proton and Photon Therapy-Induced Hematologic Toxicity in Patients With Medulloblastoma | INTERNATIONAL JOURNAL OF RADIATION ONCOLOGY BIOLOGY PHYSICS | 2021 | 38 | 7.6 |
| 90 | Uptake of 18F-fluorocholine, 18F-fluoro-ethyl-L: -tyrosine and 18F-fluoro-2-deoxyglucose in F98 gliomas in the rat | EUROPEAN JOURNAL OF NUCLEAR MEDICINE AND MOLECULAR IMAGING | 2006 | 38 | 1.9 |
| 91 | Assessment of early toxicity and response in patients treated with proton and carbon ion therapy at the Heidelberg ion therapy center using the raster scanning technique | INTERNATIONAL JOURNAL OF RADIATION ONCOLOGY BIOLOGY PHYSICS | 2011 | 37 | 2.47 |
| 92 | Radiation dose to the lens during craniospinal irradiation - An improvement in proton radiotherapy technique | INTERNATIONAL JOURNAL OF RADIATION ONCOLOGY BIOLOGY PHYSICS | 2008 | 37 | 2.06 |
| 93 | Novel technique of craniospinal axis proton therapy with the spot-scanning system - Avoidance of patching multiple fields and optimized ventral dose distribution | STRAHLENTHERAPIE UND ONKOLOGIE | 2007 | 37 | 1.95 |
| 94 | Proton FLASH Radiation Therapy and Immune Infiltration: Evaluation in an Orthotopic Glioma Rat Model | INTERNATIONAL JOURNAL OF RADIATION ONCOLOGY BIOLOGY PHYSICS | 2023 | 35 | 11.67 |
| 95 | Microdosimetry of a therapeutic proton beam with a mini-TEPC and a MicroPlus-Bridge detector for RBE assessment | PHYSICS IN MEDICINE AND BIOLOGY | 2020 | 35 | 5.83 |
| 96 | First proton minibeam radiation therapy treatment plan evaluation | SCIENTIFIC REPORTS | 2020 | 35 | 5.83 |
| 97 | Relative Biological Effectiveness Uncertainties and Implications for Beam Arrangements and Dose Constraints in Proton Therapy | SEMINARS IN RADIATION ONCOLOGY | 2018 | 35 | 4.38 |
| 98 | Supine craniospinal irradiation in pediatric patients by proton pencil beam scanning | RADIOTHERAPY AND ONCOLOGY | 2017 | 35 | 3.89 |
| 99 | A case report of pseudoprogression followed by complete remission after proton-beam irradiation for a low-grade glioma in a teenager: the value of dynamic contrast-enhanced MRI | RADIATION ONCOLOGY | 2010 | 35 | 2.19 |
| 100 | Proton Therapy for Pediatric Ependymoma: Mature Results From a Bicentric Study | INTERNATIONAL JOURNAL OF RADIATION ONCOLOGY BIOLOGY PHYSICS | 2021 | 34 | 6.8 |

TC: total citations; AC/Y: average citations per year.
